# Supplementary material for: Antiplatelet Therapy during the First Year after Acute Coronary Syndrome in a Contemporary Italian Community of over 5 Million Subjects
Source: J Clin Med. 2022 Aug 20;11(16):4888. doi: 10.3390/jcm11164888 (PMC9410031; doi:10.3390/jcm11164888)
Supplement: Supplementary file 1 [file jcm-11-04888-s001.zip › jcm-1839463-supplementary.pdf]

Figure S1 - Age distribution of the 2015 ReS database population compared to that of the Italian Institute of Statistics (ISTAT)

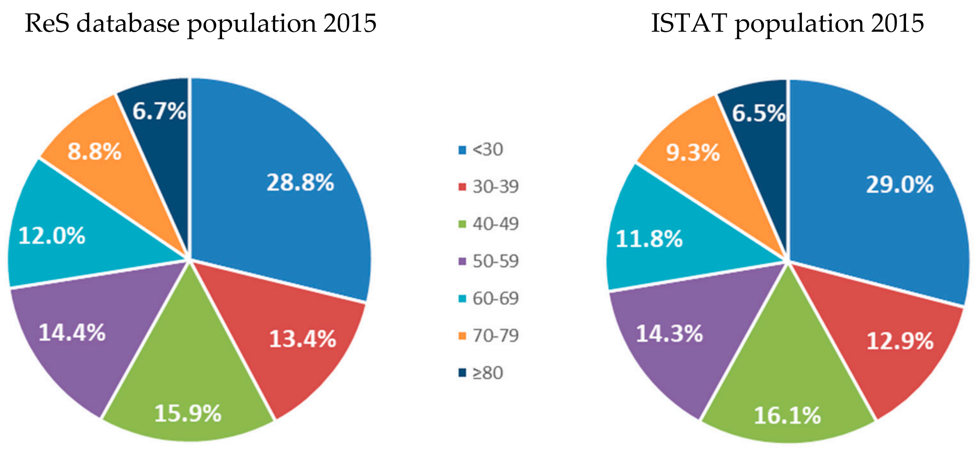

Table S1 – Criteria used to identify comorbidities prior to index date. Patients had to meet at least one criterion per comorbidity

Depression	
**Administrative database**	**Description**
**Hospitalizations**	Hospitalization with one of the following primary/secondary diagnoses (ICD-9-CM code):<br>296.2x - Major depressive disorder single episode<br>296.3x - Major depressive disorder recurrent episode<br>296.5x - Bipolar disorder, most recent episode (or current) depressed<br>296.82 - Atypical depressive disorder<br>298.0x - Depressive type psychosis<br>300.4 - Dysthymic disorder<br>301.12 - Chronic depressive personality disorder
**Disease waiver claim**	One of the following disease waiver claim code:<br>044.296.2 - Psychosis (major depressive disorder single episode)<br>044.296.3 - Psychosis (major depressive disorder recurrent episode)<br>044.296.5 - Psychosis (bipolar disorder, most recent episode (or current) depressed)<br>044.296.8 - Psychosis (manic depressive psychosis, other unspecified)<br>044.298.0 – Psychosis (depressive type psychosis)
**Pharmaceuticals**	Prescription of at least a specific drug (ATC code):<br>N06A– Antidepressants
**Diabetes**	
**Administrative database**	**Description**
**Hospitalizations**	Hospitalization with the following primary/secondary diagnosis (ICD-9-CM code):<br>250.x - Diabetes mellitus
**Disease waiver claim**	The following disease waiver claim code:<br>013 - Diabetes mellitus
**Pharmaceuticals**	A prescription of a specific drug (ATC code):<br>A10 - Drugs used in diabetes<br>AND/OR<br>A drug prescription with disease waiver claim code 013
**Dyslipidaemia**	
**Administrative database**	**Description**
**Hospitalizations**	Hospitalization with the following primary/secondary diagnosis (ICD-9-CM code):

	272.x - Disorders of lipid metabolism
**Disease waiver claim**	One of the following disease waiver claim code:<br>025 - Type IIa and IIb heterozygous familial hypercholesterolemia – Polygenic hypercholesterolemia – Familial combined hypercholesterolemia – Type III hyperlipoproteinemia
**Pharmaceuticals**	Prescription of at least 3 packs of specific drugs:<br>C10A - Lipid modifying agents, plain<br>C10B - Lipid modifying agents, combinations<br>AND/OR<br>A prescription with the disease waiver claim code 025
**Arterial hypertension**	
**Administrative database**	**Description**
**Hospitalizations**	Hospitalization with one of the following primary/secondary diagnoses (ICD-9-CM code):<br>401.x - Essential hypertension<br>402.x - Hypertensive heart disease<br>403.x - Hypertensive chronic kidney disease<br>404.x - Hypertensive heart and chronic kidney disease<br>405.x - Secondary hypertension
**Disease waiver claim**	One of the following disease waiver claim code:<br>031 – Arterial hypertension<br>A31 - Arterial hypertension without organ damage
**Pharmaceuticals**	Prescription of at least 4 packs of one or more specific drugs (ATC code):<br>C02 – Antihypertensive<br>C03 - Diuretics<br>C07 – Beta blocking agents<br>C08 – Calcium channel blockers<br>C09 – Agents acting on the renin-angiotensin system<br>AND/OR<br>A prescription with the disease waiver claim code 031 or A31
**Neoplasia**	
**Administrative database**	**Description**
**Hospitalizations**	Hospitalization with a primary/secondary diagnosis among the following (ICD-9-CM code):<br>From 140.x to 208.x - Neoplasms<br>V10.x - Personal history of malignant neoplasm<br>V58.1x – Chemotherapy<br>AND/OR<br>Hospitalization with a procedure among the following (ICD-9-CM code):

	00.10 – Implantation of chemotherapeutic agent<br>99.25 – Injection or infusion of cancer chemotherapeutic substance<br>99.28 - Injection or infusion of biological response modifier [BRM] as an antineoplastic agent
**Outpatient specialist services**	One of the following outpatient specialist services:<br>99.25 - Injection or infusion of cancer chemotherapeutic substance<br>99.24.1 – Infusion of hormonal substances
**Disease waiver claim**	The following disease waiver claim code:<br>048 – Patients affected by malignant neoplasms and by tumors of uncertain behaviour
**Pharmaceuticals**	A prescription of at least a specific drug:<br>L01 – Antineoplastic agents<br>AND/OR<br>A prescription with the disease waiver claim code 048

**Chronic obstructive pulmonary disease (COPD)/Asthma**	
**Administrative database**	**Description**
**Hospitalizations**	Hospitalization with one of the following main/secondary diagnoses (ICD-9-CM code):<br>490.x - Bronchitis, not specified as acute or chronic<br>491.x - Chronic bronchitis<br>492.x - Emphysema<br>493.x - Asthma<br>494.x - Bronchiectasis<br>496.x - Chronic airway obstruction, not elsewhere classified<br>518.81- 518.84 - Respiratory failure
**Disease waiver claim**	One of the following disease waiver claim code:<br>024 – Chronic respiratory failure<br>007 - Asthma
**Pharmaceutical**	Prescription of at least 3 packs of drugs for obstructive airway diseases:<br>R03 – Drugs for obstructive airway diseases<br>AND/OR<br>Prescription with the disease waiver claim code 024 or 007

Table S2 - Prescriptions of antithrombotic agents other than APT (oral anticoagulants and/or heparin-fondaparinux) at one month after index date

Antithrombotic agents other than APT	Patients with ACS (discharged alive) in 2017 (*n*=7966)	
	Patients treated with APT (*n*=6790)	Patients untreated with APT (*n*=1176)
Heparin and fondaparinux, n (%)	402 (6)	116 (10)
Direct oral anticoagulant, n (%)s	298 (4)	97 (8)
Vitamin K antagonist, n (%)	213 (3)	62 (5)
Total*, n (%)	807 (12)	253 (22)

*Some antithrombotic agent has been supplied more than once, thus the sum of the single items exceeds the column totals.

ACS: acute coronary syndrome; APT: antiplatelet therapy.
